# Choose Your Weapon: Defensive Behavior Is Associated with Morphology and Performance in Scorpions

**DOI:** 10.1371/journal.pone.0078955

**Published:** 2013-11-13

**Authors:** Arie van der Meijden, Pedro Lobo Coelho, Pedro Sousa, Anthony Herrel

**Affiliations:** 1 CIBIO, Centro de Investigação em Biodiversidade e Recursos Genéticos, Campus Agrário de Vairão, Vairão, Portugal; 2 UMR 7179, Muséum National d′Histoire Naturelle, Département d′Ecologie et de Gestion de la Biodiversité, Paris, France; CNRS, France

## Abstract

Morphology can be adaptive through its effect on performance of an organism. The effect of performance may, however, be modulated by behavior; an organism may choose a behavioral option that does not fully utilize its maximum performance. Behavior may therefore be decoupled from morphology and performance. To gain insight into the relationships between these levels of organization, we combined morphological data on defensive structures with measures of defensive performance, and their utilization in defensive behavior. Scorpion species show significant variation in the morphology and performance of their main defensive structures; their chelae (pincers) and the metasoma (“tail”) carrying the stinger. Our data show that size-corrected pinch force varies to almost two orders of magnitude among species, and is correlated with chela morphology. Chela and metasoma morphology are also correlated to the LD50 of the venom, corroborating the anecdotal rule that dangerously venomous scorpions can be recognized by their chelae and metasoma. Analyses of phylogenetic independent contrasts show that correlations between several aspects of chela and metasoma morphology, performance and behavior are present. These correlations suggest co-evolution of behavior with morphology and performance. Path analysis found a performance variable (pinch force) to partially mediate the relationship between morphology (chela aspect ratio) and behavior (defensive stinger usage). We also found a correlation between two aspects of morphology: pincer finger length correlates with the relative “thickness” (aspect ratio) of the metasoma. This suggests scorpions show a trade-off between their two main weapon complexes: the metasoma carrying the stinger, and the pedipalps carrying the chelae.

## Introduction

Behavior, i.e. the response of an animal when faced with behavioral options [1], is often viewed as an important driver of evolutionary diversification. Some authors have argued that behavioral flexibility may also constrain phenotypic evolution [2]. The fact that animals can use different behaviors depending on the context may blur the relationships between morphology, performance and ecology [1], [3], [4]. Consequently, selection on performance capacity may be decoupled from ecology [4], and behavioral variation can result in a many-to-one mapping of performance on ecology [5]. On the other hand, links between behavior, performance, and morphology have been demonstrated at the inter- and intra-specific level [6]–[10]. Moreover, behavioral traits can have a genetic basis and as such can be under direct selection [11], [12]. Yet, which aspects of morphology and performance are related to behavior and whether morphology and performance variation constrains or enhances the evolution of different behaviors, or *vice versa*, remains poorly understood. Given that behavioral traits typically show less phylogenetic signal [13], the evolution of behavior may indeed be decoupled from the evolution of morphology and performance for many functions. In active defensive behavior against predators; however, the prey’s fitness is maximized by successfully deterring the predator. The arms race between the prey’s deterrence capacity and the predator’s ability to withstand or circumvent these defenses requires the prey to select its maximum performance in the context of its defensive behavior. It is therefore likely that defensive behavior against predators, which we study here, is more closely correlated to maximum performance than behaviors for which non-maximum performance also has a fitness benefit [4].

Occurring worldwide in terrestrial habitats ranging from temperate forest to deserts and tropical forests, scorpions have ecologically diversified considerably, with nearly 2000 described species, and many more cryptic species are awaiting discovery [14], [15]. Several ecomorphotypes based on relative sizes of specific body parts have been qualitatively described [16], [17]. A large part of the morphological variation of scorpions resides in their most emblematic body parts; the pincers or chelae [18], and the tail-like metasoma carrying the venomous stinger. These structures are used in defense [19], as well as in prey capture and incapacitation. Scorpions can form a large part of the animal biomass in some habitats [17], and are therefore an important food resource for some predators. Scorpions possess defensive responses that elicit fear in mammals [20]. Some predators, however, such as Hemprich’s long-eared bat (*Otonycteris hemprichii*), have developed insensitivity to scorpion defenses in order to utilize this important resource [21]. Most scorpions will avoid contact with predators by retreating to a burrow or other hiding place. When cornered or apprehended by a predator, a scorpion can choose to use either its chelae or its venomous telson (stinger), or both. The distribution of the defensive capacities of scorpions between the chelae and the telson may results in an evolutionary trade-off in the investment in these two systems; some species have developed powerful chelae and others have a well-developed metasoma carrying the venomous stinger. In interspecific interactions, scorpion species with larger chelae are known to use them more, whereas the Buthidae, having more slender chelae, use their metasoma more in defense [22]. In fact, the relative size of chelae and metasoma is often used as a rule of thumb to assess whether an unknown scorpion may be dangerously venomous or not [23]–[25]. The species with more robust chelae produce a much higher pinch force [26], and finite element analyses show that their low-aspect ratio shapes allow cuticular stresses to remain lower during application of maximum forces, making them better suited for use in defense [18]. The “bite” force of the chelae of scorpions relative to their body mass is highly variable, and spans a range of almost three orders of magnitude [27], suggesting this performance variable may be subject to differential selection for its different functions (such as prey prehension, mating, sensing, defense etc.), although neutral variation cannot be excluded.

In other groups important evolutionary connections have been observed in the relationships between venom compounds and the evolution of the venom gland. For example, in squamates [28] the structure of the delivery system [29] and its functional performance [30] are intimately associated with the evolution of the venoms themselves [31]. The evolution of the venom, and its mechanical delivery system, are therefore intimately related and must be studied together. We here present comparative data on the association between the morphology and performance of the defensive structures of different species of scorpions, and their defensive behavior. Our data show a large variety in defensive behaviors, and an evolutionary association with both morphology and performance.

## Materials and Methods

### Ethics Statement

Buthid scorpions were kept under ICNB license 05/2010/CAPT. No additional permits were required for the described experimental manipulations. When necessary, subjects were anesthetized using Isoflurane. All efforts were made to minimize suffering.

### Taxon Selection and Animal Maintenance

A total of 26 scorpion species were selected to represent a broad range of chela and telson morphologies based on their availability (Table 1). All specimens were procured from the pet trade, and kept in captivity for at least several weeks before experiments commenced. Species were identified using specific keys [17], [32]–[40].

**Table 1:** Species and numbers of specimens used for each aspect of the study.

		Number of specimens				Genbank and Datadryad accession numbers		
Species name	Family	Force	Morphology	Mech. Adv.	Behavior	12S	16S	CO1
*Androctonus amoreuxi*	Buthidae	8	8	8	12	JQ423120	JQ514228	JQ514246
*Androctonus liouvillei*	Buthidae	0	4	0	6	KF548106	doi:10.5061/dryad.7r4p9	n.a.
*Androctonus bicolor*	Buthidae	0	2	0	9	KF548109	n.a.	KF548120
*Buthacus sp.*	Buthidae	0	3	0	11	KF548102	doi:10.5061/dryad.7r4p9	KF548116
*Buthus lienhardi*	Buthidae	11	11	11	11	KF548097	doi:10.5061/dryad.7r4p9	KF548110
*Buthus cf. paris*	Buthidae	6	6	6	6	KF548098	doi:10.5061/dryad.7r4p9	KF548111
*Buthus draa*	Buthidae	6	6	6	6	KF548099	doi:10.5061/dryad.7r4p9	KF548112
*Buthus mariefrance*	Buthidae	0	3	0	6	n.a.	doi:10.5061/dryad.7r4p9	n.a.
*Grosphus flavopiceus*	Buthidae	13	11	0	8	JQ423127	JQ514238	JQ514254
*Hottentota gentili*	Buthidae	10	10	10	11	JQ423119	JQ514227	JQ514245
*Hottentotta trilineata*	Buthidae	0	3	0	3	n.a.	n.a.	n.a.
*Leiurus quinquestriatus*	Buthidae	9	9	5	4	JQ423131	JQ514241	JQ514258
*Orthochirus innesi*	Buthidae	0	3	0	7	JQ423118	JQ514226	JQ514244
*Parabuthus transvaalicus*	Buthidae	4	5	5	11	JQ423121	JQ514229	JQ514247
*Euscorpius flavicaudus*	Euscorpiidae	0	3	0	13	KF548103	JQ514237	KF548117
*Hadogenes cf paucidens*	Liochelidae	13	12	4	9	JQ423130	doi:10.5061/dryad.7r4p9	JQ514257
*Iomachus politus*	Liochelidae	8	8	0	10	KF548108	doi:10.5061/dryad.7r4p9	KF548119
*Opisthacanthus asper*	Liochelidae	8	8	0	6	KF548107	doi:10.5061/dryad.7r4p9	KF548118
*Opisthacanthus madagascariensis*	Liochelidae	7	7	0	9	KF548105	JQ514236	n.a.
*Caraboctonus keyserlingi*	Iuridae	9	9	1	10	JQ423123	JQ514231	JQ514249
*Hadrurus arizonensis*	Iuridae	9	9	5	8	JQ423129	JQ514240	JQ514256
*Hetrometrus laoticus*	Scorpionidae	11	11	11	11	KF548100	doi:10.5061/dryad.7r4p9	KF548113
*Opistophthalmus boehmi*	Scorpionidae	6	6	1	12	KF548104	doi:10.5061/dryad.7r4p9	JQ514248
*Pandinus imperator*	Scorpionidae	14	10	8	9	n.a.	JQ514234	KF548115
*Scorpio fuliginosus*	Scorpionidae	8	8	8	8	JQ423132	JQ514242	JQ514259
*Scorpio maurus*	Scorpionidae	0	6	0	15	n.a.	n.a.	n.a.
					**231**			

All animals were kept under species-specific circumstances [26] and appeared in good health throughout the test period and beyond. Desert species (*Androctonus, Buthacus, Buthus, Hadogenes, Hadrurus, Hottentotta, Leiurus, Orthochirus, Parabuthus,* and *Scorpio*) were kept in plastic boxes (123×190×80 mm for small species; 200×230×130 mm for larger species) on a layer of ground cork substratum. Species requiring more humid circumstances (*Caraboctonus, Euscorpius, Grosphus Hetrometrus, Iomachus, Opisthacanthus, Opistophthalmus* and *Pandinus*) were kept in plastic boxes with humid substrate and sprayed with water regularly. All animals were fed with crickets (*Acheta* sp.) and cockroaches (*Blaptica* sp.) once every 1–2 weeks before and during the experiment. All specimens were provided with a piece of polyethylene tubing as a hiding place, and kept at 24°−26°C. Although optimum temperatures for the species in this study are not known, the used maintenance conditions were chosen as all species have been kept under these conditions in good health for several years by one of us (AvdM). Data from specimens that died, gave birth or molted during the study were excluded. From a small subsample a haemolymph smear was inspected for the presence of parasites after the test period, and none were discovered.

### Behavioral Trials

**Figure 1:**
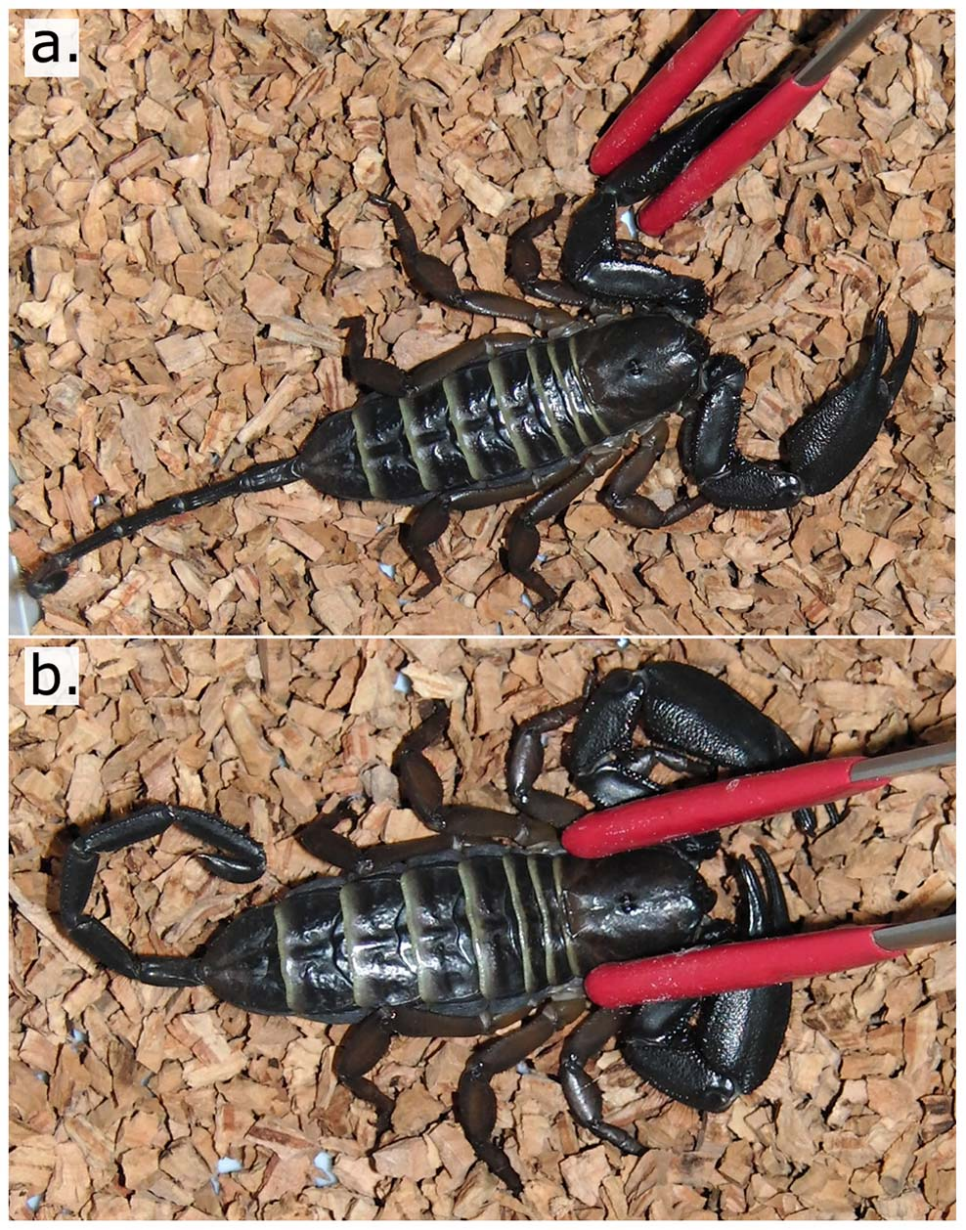
Defensive behavior trials, shown on a specimen of *Hadogenes* cf. *paucidens*. First each chela is pinned to the ground using rubber-tipped forceps for 5 seconds (a.). Subsequently, the prosoma is pinned down for 5 seconds (b.), and the defensive response categorized as using one or both chelae (1), the telson (2), chelae and telson (3) or neither (0).

Behavioral trials were executed to estimate qualitative differences in the defensive response of scorpions. Before the trial proper was started, the scorpion was aroused by gently tapping the pedipalps and/or prosoma until an alert posture was assumed (chelae extended and metasoma erect). Each trial consisted of first restraining each of the chelae in arbitrary order for five seconds using large rubber-tipped tweezers, followed by a similar restriction of the prosoma (figure 1). Each trial therefore resulted in three behavioral responses. Two responses from restraining each of the two chelae, and one from restraining the prosoma. Only actual gripping motion on the tweezers and directed stinging were scored as defensive behaviors. All behavioral trials were performed in the enclosure of the scorpion. Each specimen was subjected to these behavioral trials five times, spaced by at least one day. To allow a comparative study, and as optimum temperatures are unknown for most of the included species, we chose to make all behavioral and performance observations at standardized environmental conditions. All behavior trials were performed by a single person (AvdM) in a climate controlled room at 23-24°C. This temperature range was chosen as both tropical and desert species have been observed to be active at these temperatures. This temperature range is on the low end of the temperature range to which all specimens were acclimated for several weeks before the experiments started, thus mimicking the nighttime conditions during which scorpions are normally active. Behavioral responses were scored in the following categories: (0) none; (1) chelae only; (2) telson only; (3) Both chelae and telson. For further analysis, except where stated otherwise, the responses from the chela restrictions and the prosoma restrictions were pooled. We also calculated the proportions of the active responses without the non-responses (0), in order to quantify what a scorpion uses in response if it chooses to respond at all (designated if1, if2 and if3).

For visualization of the data, hierarchical cluster analysis was performed using Euclidian distances. We also clustered the species based on active responses only (if1, if2, if3), and on the proportion of the usage of chelae (category 1+3; TotC) and telson (category 2+3; TotT) in defense. We performed non-parametric Fisher's exact tests on the behavioral data in order to identify significant differences in behavior among species (Table 2). In addition, a Fisher’s exact test was used to test for differences in behavioral responses to chela restriction versus prosoma restriction. These statistical analyses were performed in R [41].

**Table 2:** Differences in defensive responses between species as tested with a two-sided Fisher's exact test.

species	Family	#	1	2	3	4	5	6	7	8	9	10	11	12	13	14	15	16	17	18	19	20	21	22	23	24	25	26
*Androctonus amoreuxi*	Buthidae	1		**0.096**	0.003	3.2E-12	1.3E-04	5.5E-05	3.7E-05	**0.552**	**0.487**	**0.290**	**0.184**	**0.484**	**0.560**	1.7E-04	2.2E-11	9.4E-12	3.9E-20	2.4E-05	9.8E-17	6.8E-09	8.7E-08	1.0E-14	1.2E-12	1.3E-15	4.6E-12	2.5E-15
*A. liouvillei*	Buthidae	2	**1**		**0.482**	2.6E-11	8.0E-06	3.1E-06	1.6E-06	**0.083**	**0.338**	**0.017**	**0.731**	**0.075**	**0.610**	**0.229**	4.8E-11	2.2E-12	9.7E-18	2.1E-06	7.0E-15	4.6E-09	1.8E-08	3.7E-13	1.7E-11	4.7E-14	1.3E-11	5.2E-13
*A. bicolor*	Buthidae	3	**1**	**1**		3.6E-19	4.9E-10	3.6E-10	1.3E-10	8.0E-03	**0.043**	1.4E-04	**1**	3.2E-03	**0.183**	**0.661**	6.7E-18	1.0E-17	4.2E-27	1.1E-07	1.6E-23	3.4E-15	9.5E-14	1.7E-21	1.5E-19	1.5E-22	8.4E-19	3.2E-22
*Buthacus* sp.	Buthidae	4	**1**	**1**	**1**		0.003	**0.093**	**0.085**	9.6E-04	1.3E-11	5.8E-09	2.2E-05	1.8E-04	8.4E-07	4.6E-24	**0.785**	**0.027**	5.1E-04	**0.019**	**0.025**	**0.275**	**0.392**	**0.369**	**1**	**0.112**	**0.727**	**0.657**
*Buthus lienhardi*	Buthidae	5	**1**	**1**	**1**	**1**		**0.494**	**0.442**	**0.128**	4.9E-05	6.5E-03	2.2E-03	**0.072**	2.1E-03	6.5E-13	2.3E-03	3.8E-05	4.9E-09	1.9E-03	1.7E-06	**0.064**	**0.078**	1.3E-04	2.3E-03	1.8E-05	1.3E-03	2.3E-04
*B. cf. paris*	Buthidae	6	**1**	**1**	**1**	**1**	**1**		**1**	**0.051**	2.1E-05	2.2E-03	1.3E-03	**0.026**	9.7E-04	1.2E-12	**0.070**	1.3E-03	1.3E-05	**4.6E-03**	6.0E-04	**0.448**	**0.412**	**0.015**	**0.078**	**4.0E-03**	**0.048**	**0.028**
*B. draa*	Buthidae	7	**1**	**1**	**1**	**1**	**1**	**1**		**0.054**	1.2E-05	1.4E-03	8.0E-04	**0.027**	6.7E-04	2.8E-13	**0.062**	1.5E-03	8.0E-06	**4.5E-03**	5.5E-04	**0.404**	**0.424**	**0.011**	**0.072**	2.7E-03	**0.043**	**0.027**
*B. mariefrance*	Buthidae	8	**1**	**1**	**1**	**1**	**1**	**1**	**1**		**0.284**	**1**	**0.118**	**1**	**0.313**	1.7E-03	6.6E-04	1.6E-05	3.4E-07	3.3E-04	8.5E-06	**6.7E-03**	**7.8E-03**	1.5E-04	9.8E-04	5.0E-05	7.0E-04	3.7E-04
*Grosphus flavopiceus*	Buthidae	9	**1**	**1**	**1**	**1**	**1**	**1**	**1**	**1**		**0.107**	**0.375**	**0.248**	**0.831**	**6.0E-03**	4.4E-11	5.9E-12	7.7E-19	1.0E-05	1.2E-15	8.6E-09	6.4E-08	1.2E-13	6.4E-12	1.4E-14	1.0E-11	9.1E-14
*Hottentota gentili*	Buthidae	10	**1**	**1**	**1**	**1**	**1**	**1**	**1**	**1**	**1**		**0.079**	**1**	**0.199**	3.9E-06	2.2E-08	2.8E-09	3.8E-16	1.0E-04	5.6E-13	4.4E-06	1.8E-05	6.8E-11	3.4E-09	6.9E-12	5.0E-09	4.1E-11
*H. trilineata*	Buthidae	11	**1**	**1**	**1**	**1**	**1**	**1**	**1**	**1**	**1**	**1**		**0.138**	**0.487**	**0.687**	1.5E-05	3.2E-07	9.7E-09	1.3E-05	3.4E-07	1.3E-04	1.9E-04	3.1E-06	1.3E-05	7.9E-07	8.8E-06	5.1E-06
*Leiurus quinquestriatus*	Buthidae	12	**1**	**1**	**1**	**1**	**1**	**1**	**1**	**1**	**1**	**1**	**1**		**0.252**	6.3E-04	1.5E-04	2.8E-06	1.1E-08	2.3E-04	6.1E-07	2.8E-03	2.8E-03	1.7E-05	1.9E-04	4.4E-06	1.1E-04	3.2E-05
*Orthochirus innesi*	Buthidae	13	**1**	**1**	**1**	**1**	**1**	**1**	**1**	**1**	**1**	**1**	**1**	**1**		**0.089**	1.2E-06	1.5E-08	1.7E-11	2.0E-05	2.0E-09	3.2E-05	5.0E-05	6.6E-08	8.2E-07	1.2E-08	4.5E-07	1.4E-07
*Parabuthus transvaalicus*	Buthidae	14	**1**	**1**	**1**	**1**	**1**	**1**	**1**	**1**	**1**	**1**	**1**	**1**	**1**		3.0E-22	9.6E-21	1.1E-32	2.3E-08	1.5E-28	1.9E-19	4.1E-17	6.5E-27	7.8E-25	9.7E-28	1.5E-23	3.4E-28
*Euscorpius flavicaudus*	Euscorpiidae	15	**1**	**0.203**	**1**	**0.590**	**0.040**	**0.2069**	**1**	1.6E-04	**1**	**1**	**0.041**	**0.203**	9.0E-04	**1**		**0.074**	**4.2E-03**	**3.6E-02**	**0.081**	**0.188**	**0.297**	**0.646**	**0.788**	**0.281**	**1**	**1**
*Hadogenes cf paucidens*	Liochelidae	16	**1**	**0.205**	**1**	**0.591**	**0.041**	**0.2091**	**1**	2.4E-04	**1**	**1**	**0.043**	**0.205**	1.2E-03	**1**	**1**		**0.771**	**1.6E-01**	**0.575**	**5.2E-03**	**8.0E-03**	**0.115**	**0.028**	**0.279**	**0.060**	**0.044**
*Iomachus politus*	Liochelidae	17	**0.106**	3.0E-05	0.012	1.5E-03	2.3E-08	1.2E-05	**0.106**	1.1E-18	**1**	0.012	4.3E-09	3.0E-05	4.1E-18	**0.323**	1.4E-11	1.8E-12		**2.8E-01**	**0.271**	1.9E-05	1.3E-04	**8.1E-03**	5.8E-04	**0.075**	4.0E-03	1.4E-03
*Opisthacanthus asper*	Liochelidae	18	**1**	**1**	**1**	**1**	0.347	**0.5927**	**1**	**0.025**	**1**	1	**0.347**	1	**0.091**	**1**	**0.059**	**0.045**	3.6E-17		**0.090**	**1.2E-02**	**0.013**	**0.040**	**0.019**	**0.082**	**0.036**	**0.038**
*O. madagascariensis*	Liochelidae	19	**0.146**	1.6E-04	0.024	0.004	6.9E-07	7.6E-05	**0.146**	8.8E-15	**1**	0.024	1.5E-07	1.6E-04	5.0E-14	**0.377**	6.1E-08	2.8E-08	**0.599**	1.6E-12		2.0E-03	**5.8E-03**	**0.156**	**0.027**	**0.545**	**0.091**	**0.053**
*Caraboctonus keyserlingi*	Iuridae	20	**1**	**1**	**1**	**1**	**1**	**1**	**1**	**1**	**1**	1	**1**	**1**	**1**	**1**	**1**	**1**	**0.106**	**1**	**0.146**		**1**	**0.050**	**0.249**	**0.011**	**0.174**	**0.102**
*Hadrurus arizonensis*	Iuridae	21	**1**	**0.291**	**1**	**0.5526**	**0.060**	**0.1567**	**1**	2.2E-03	**1**	1	**0.053**	**0.291**	**7.9E-03**	**1**	**1**	**1**	4.0E-05	**1.5E-01**	5.9E-04	**1**		**0.102**	**0.398**	**0.026**	**0.267**	**0.208**
*Hetrometrus laoticus*	Scorpionidae	22	0.040	3.9E-06	2.6E-03	2.3E-04	1.4E-08	1.6E-06	0.040	7.6E-16	**1**	2.6E-03	1.9E-09	3.9E-06	4.7E-15	**0.185**	1.7E-09	7.9E-10	**0.139**	2.7E-13	**0.073**	0.040	1.1E-05		**0.378**	**0.475**	**0.660**	**0.598**
*Opistophthalmus boehmi*	Scorpionidae	23	**0.467**	**0.023**	**0.208**	**0.085**	0.005	**0.018**	**0.467**	1.3E-04	**1**	**0.208**	3.4E-03	**0.023**	4.9E-04	**1**	**0.070**	**0.065**	**0.434**	7.5E-03	**0.470**	**0.467**	**0.176**	**0.066**		**0.100**	**0.732**	**0.664**
*Pandinus imperator*	Scorpionidae	24	**0.105**	6.8E-05	0.013	2.0E-03	2.5E-07	3.1E-05	**0.105**	2.9E-14	**1**	0.013	8.7E-08	6.8E-05	1.8E-13	**0.316**	8.7E-08	6.6E-08	**1**	9.7E-12	**0.517**	**0.105**	2.2E-04	**0.238**	**0.410**		**0.258**	**0.189**
*Scorpio fuliginosus*	Scorpionidae	25	**1**	**0.125**	**0.467**	**0.455**	**0.054**	**0.1103**	**1**	**8.8E-03**	**1**	**0.467**	**0.046**	**0.125**	**0.022**	**1**	**0.600**	**0.599**	**0.175**	**0.119**	**0.202**	**1**	**0.603**	2.3E-02	**0.627**	**0.161**		**1**
*S. maurus*	Scorpionidae	26	**0.4**	**0.011**	**0.143**	**0.033**	0.003	8.8E-03	**0.4**	1.4E-04	**1**	**0.143**	2.0E-03	**0.011**	4.4E-04	**0.400**	**0.033**	**0.030**	**1**	4.7E-03	**1**	**0.400**	**0.058**	**0.538**	**0.576**	**1**	**0.524**	

A sequential Bonferroni correction has been applied with an initial alpha of 0.05, and P-values higher than alpha, indicating no significant difference between the species, are shown in bold. Values below the diagonal take into account all possible defensive responses including no response (see table 1). Values above the diagonal indicate differences in chela and telson use in defense. Significant differences in the defensive responses between many of the studied species were found. Species of the family Buthidae cannot be distinguished based on their combined defensive responses. When looking at only chela or telson use however, there are several significant differences between species pairs within the Buthidae. Remarkable is that both *Grosphus flavopiceus* and *Parabuthus transvaalicus* cannot be distinguished from any other species in this study when taking all defensive responses into account. When just testing the chela and telson use however, they show significant differences with other species.

### Morphology

All external measurements were made using digital calipers on preserved or isoflurane anesthetized specimens. These specimens were either the specimens used in other aspects of this study, or specimens of similar size from the same source. Measurements of the distance between the fulcrum of the movable finger of the chela and the manus, and the muscle insertion point furthest away from it were either made by hand using digital calipers on the disjointed movable finger of preserved specimens, or taken from high-resolution CT or synchrotron scans [18]. These internal measurements to determine the force inlever of the movable finger were made on a subsample of the specimens available for each species, or only once when anatomical scan data were available. Because scorpions can vary considerably in length, girth and weight depending on their feeding state, we used the length of the prosoma, which does not vary between molts, as an indicator of overall size [32]. Regression of morphological variables on prosoma length as a proxy for size was not significant, as there are large differences in the relative sizes of the chelae and metasoma between species independent of overall size variation. The calculation of regression residuals was therefore not appropriate. Several linear measurements were combined in order to give functionally relevant ratios. Chela aspect ratio (AR) is the height of the chela manus divided by the total length of the chela. This ratio has been shown to be highly correlated with pinch force [26]. Similarly, metasoma AR was calculated by dividing the metasoma length by the product of the average height of the 1^st^, 3^rd^ and 5^th^ metasomal segment and the average of the width of those segments to provide a single value for metasoma girth. The ratio of the movable finger to the chela length was obtained by dividing the total length of the chela by the length of the movable finger, and captures the relative length of the chela fingers. Longer-fingered chelae will thus result in a smaller value. Since relatively longer-fingered chelae will have a longer outlever, and a reduced space for muscles, we expect that long fingers will correlate to reduced pinch performance. Mechanical advantage was calculated by dividing the average of the distance from the muscle insertion to the axis of rotation for the left and right chela with the average of the length of the movable finger for both chelae. This measure is therefore the displacement advantage, and inverse to the force advantage, and expected to be lower in species with stronger chelae. Some scorpion species have reduced metasoma lengths and relative metasoma length was obtained by dividing metasoma length by the prosomal length, the latter being a good estimate of overall size (see above). A logistic regression was carried out to identify correlations between morphological characters and behavioral variables, with the behavioral classes as the dependent variable.

### Performance Measurement

*In vivo* pinch forces were measured using either a Kistler force transducer (type 9203, Kistler Inc., Switzerland) mounted on a purpose-built holder [42], or using a similar setup using a Sauter FH20 external force sensor (Sauter ltd., Germany). Measurements were made in a climate-controlled room set at 23–24°C. During pinch-force measurements, scorpions were restrained between sponge pads in which a cutout was made to accommodate the body, or by placing a padded clamp over the last segments of the metasoma to allow safe handling. Five trials were performed, separated by at least one day. Only the maximum force per individual was retained for further analyses. In order to obtain pinch forces corrected for body size, we attempted to use a linear regression of pinch force on prosoma length across all species. As these variables did not show any linear relationship (R^2^<0.02), presumably due to the effects of chela design obscuring the effects of body size, we chose to correct for size by dividing pinch force by the square of the prosoma length. Pinch force must be scaled by prosoma length squared, as force scales with the physiological cross section of the muscle, which in turn scales with length squared [43].

The LD50 of 14 species of scorpions were included as a second defensive performance variable. Where no LD50 was available, the value of a closely related species was used given that this variable is thought to be conserved within genera (see table 3).

**Table 3:** All behavioral responses by category, proportions of each response type, number of specimens, LD50 and measured variables.

			Behavioral responses				Max.		Mech.				CL/	Max. F/	Met.	Met.	LD50	Alternate	Reference
Species name	Family	# spec.	0	1	2	3	P. force (N)	SD	Adv.	SD	AR	SD	MV	Pros. L.	Th.	L.	(mg/kg)	LD50 species	LD50
*Androctonus amoreuxi*	Buthidae	12	2	0	136	42	2.53	0.72	4.60	0,18	3.74	0,06	1.55	0.0316	1.69	4.33	0.75		[66]
*Androctonus liouvillei*	Buthidae	6	10	0	70	10					5.70	0,20	1.41		1.34	4.39	0.32	*A. mauretanicus*	[62]
*Androctonus bicolor*	Buthidae	9	4	0	120	11					5.40	0,11	1.36		0.89	4.02	0.40		[62]
*Buthacus sp.*	Buthidae	11	6	0	10	149					4.49	0,56	1.61		4.07	5.16	3.5		[62]
*Buthus lienhardi*	Buthidae	11	18	0	67	80	1.28	0.36	5.50	0,56	3.79	0,31	1.56	0.0249	1.67	4.62			
*Buthus cf. paris*	Buthidae	6	11	0	28	51	0.51	0.27	6.63	0,67	4.74	0,43	1.50	0.0159	2.44	4.87	4.15		[62]
*Buthus draa*	Buthidae	6	2	0	31	57	1.16	0.71	6.53	1,58	4.20	0,39	1.56	0.0187	1.73	4.77			
*Buthus mariefrance*	Buthidae	6	53	0	26	11					3.45	0,13	1.65		2.45	4.31			
*Grosphus flavopiceus*	Buthidae	8	0	0	97	23	1.71	1.02			3.92	0,33	1.70	0.0180	1.98	5.07			
*Hottentota gentili*	Buthidae	11	4	0	112	49	0.73	0.37	7.17	0,85	5.41	0,6	1.45	0.0111	2.15	4.57			
*Hottentotta trilineata*	Buthidae	3	20	0	23	2					3.55	0,36	1.64		1.87	4.36			
*Leiurus quinquestriatus*	Buthidae	4	10	0	35	15	0.63	0.16	7.58	0,26	5.99	0,18	1.39	0.0066	1.93	4.72	0.33		[62]
*Orthochirus innesi*	Buthidae	7	56	1	41	7					5.18	*	1.56		2.13	4.34	2.67		[67]
*Parabuthus transvaalicus*	Buthidae	11	1	0	153	11	0.5	0.28	5.59	0,84	4.42	0,98	1.52	0.0052	0.94	4.42	4.25		[62]
*Euscorpius flavicaudus*	Euscorpiidae	13	77	19	19	80					2.70	0,03	1.73		5.3	2.53			
*Hadogenes cf paucidens*	Liochelidae	9	87	21	2	25	15.13	5.44	3.82	0,14	2.93	0,14	2.02	0.0980	6.8	3.82	1800	*H. troglodytes*	[62]
*Iomachus politus*	Liochelidae	10	29	63	13	45	3.92	1.07			2.89	0,41	2.23	0.0356	9.27	2.55			
*Opisthacanthus asper*	Liochelidae	6	81	8	1	0	12.79	3.86			2.12	0,07	1.76	0.2220	1.75	1.92			
*O. madagascariensis*	Liochelidae	9	22	38	5	70	3.28	0.38			2.36	0,04	1.85	0.0582	5.05	2.44			
*Caraboctonus keyserlingi*	Iuridae	10	2	0	33	115	2.28	0.34	4.26		2.91	0,17	1.73	0.0507	1.82	2.98			
*Hadrurus arizonensis*	Iuridae	8	19	5	25	71	3.92	0.92	5.14	0,31	2.99	0,1	1.42	0.0238	1.75	3.95	198		[68]
*Hetrometrus laoticus*	Scorpionidae	11	4	22	9	130	26.36	8.81	3.25	0,11	1.95	0,11	1.76	0.1353	1.36	2.84	300	*H. longimanus*	[69]
*Opistophthalmus boehmi*	Scorpionidae	12	4	4	14	158	5.49	0.48	3.61		2.03	0,19	1.70	0.0586	2.5	2.41	430	*O. glabrifrons*	[70]
*Pandinus imperator*	Scorpionidae	9	11	26	1	97	12.96	3.75	3.44	0,22	1.66	0,09	1.63	0.0542	1.39	3.60	40	*P. exitialis*	[71]
*Scorpio fuliginosus*	Scorpionidae	8	4	2	1	113	11.16	3.13	2.93	0,16	1.56	0,07	1.76	0.1368	1.83	3.15			
*Scorpio maurus*	Scorpionidae	15	1	3	1	220					1.55	0,06					141.6		[62]
	**Sum**	**231**	**538**	**212**	**1073**	**1642**													
	%		**15.5**	**6.1**	**31.0**	**47.4**													

Max.P. force: maximum pinch force, Mech. Adv.: Mechanical Advantage of the chela lever system, AR: Aspect Ratio of the chela, CL/MV: Chela length divided by length movable finger, Max. F/Pros. L.: Maximum Force divided by Prosoma length to provide a body-size corrected value for pinch force, Met. Th.: Relative metasoma thickness, Met. L.: Relative metasoma length, SD: standard deviation of the variable in the previous column. Note that when an LD50 value was not known for a particular species, a closely related species was selected when available.

### Phylogenetic Analysis

Tissue samples were taken from specimens preserved in 96% ethanol. Muscle tissue was taken from one or more of the walking legs and digested using proteinase K (10 mg/ml concentration). DNA was extracted using a standard salt extraction protocol [44]. Fragments of the mitochondrial genes 12S, 16S were amplified using primers 12S_F_AvdM and 12s_r_AvdM [18] for 12S and LR-J-12887 [45] and a scorpion- specific primer for the heavy strand [46] for 16S. The CO1 gene fragment was amplified using LCO1490 and HCO2198 [47] or COI_avdm_F and COI_avdm_R [18]. PCR conditions differed only in annealing temperature. Cycle conditions were adapted from standard PCR protocol reactions with an initial denaturation step at 94°C for 3 min, followed by 35 cycles with 94°C for 30 s, a primer pair specific annealing temperature during 45s and extending for 1 min at 72°C. The final extension was carried out at 72°C for 5 min. Annealing temperatures were 48°C-49°C for CO1, 50°C for 16S and 52°C for 12S. The PCR products were purified and sequenced using dye-labeled dideoxy terminator cycle at a commercial sequencing company (Macrogen inc.) using the corresponding PCR primers. Chromatograms were checked using FinchTV, version 1.4.0 (Geospiza, Inc., USA; http://www.geospiza.com). The obtained sequences were aligned using MEGA 5 [48]. The coding sequence of CO1 was aligned based on the translated amino acid sequence. 12S and 16S rRNA sequences were aligned using Muscle [49] as incorporated into MEGA 5 using the default settings. The best fitting model of molecular evolution was found to be GTR+I+G using JModeltest [50]. The full alignment was then used to produce a ML estimate of the branch lengths with MEGA 5 [48]. We restrained the phylogenetic analysis to group members of the same family together based on the taxonomy provided by Prendini & Wheeler [51]. Since phylogenetic reconstruction based on the combined alignment did not resolve all relationships with high support, we introduced three polytomies where the phylogeny was incongruous with current taxonomy. One polytomy was introduced at the base of the clade uniting all scorpionids, one at the base of the clade uniting *Grosphus*, *Buthacus*, *Parabuthus* and *Leiurus*, and one more polytomy at the base of the clade uniting the previous clade and that uniting all *Androctonus* specimens. The branch lengths were transformed to obtain an ultrametric phylogram, which was used to calculate phylogenetic independent contrasts using the PDAP package [52], [53] inMesquite [54]. A Brownian motion model of evolution was employed. Two species of *Buthus* were found to be connected by a very short branch, causing extreme contrast values. One of the species, *Buthus paris*, was therefore arbitrarily chosen and removed from the independent contrasts analysis. All performance and morphological variables were log10 transformed prior to analysis. All regression analyses were performed on the average values per species. The behavioral data was converted to proportions per species, and all variables arcsin transformed, except for variables 1 and if1, for which a square root transformation was needed to obtain a normal distribution. We used the diagnostics provided in the PDAP program implemented in Mesquite to test whether branch lengths were indeed appropriate for the data used. Regressions between the standardized contrasts were run through the origin to test for co-evolution between traits across the tree. As we considered the polytomy as a soft polytomy we subtracted one degree of freedom for each polytomy [55].

**Figure 2:**
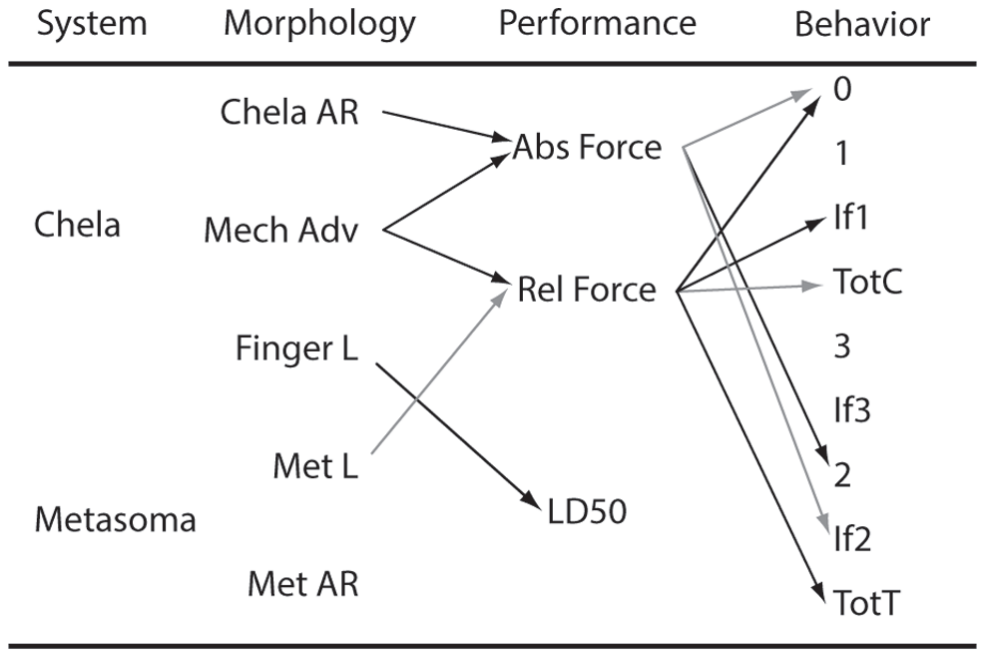
Schematic of the significant (black) correlations between variables. Correlations which with p-values between 0.05 and 0.1 are shown in grey. Each well-supported path between morphological, performance and behavioral variables were tested using Sobel’s test of mediation. Of the six paths tested, only the path between chela aspect ratio, maximum force and behavioral category 2 (stinger use) displayed partial mediation of the performance variable (p = 0.026). Two more paths were found to show some indication of mediation (Mech.Adv - Rel. Force - IF1, p = 0.088; Mech. Adv. – Rel. Force – TotT, p = 0.069).

In the paradigm of functional morphology, morphology does not affect behavior directly, but rather trough a mediation effect of performance [1]. We therefore tested for mediation effects of performance variables on the relationship between morphological variables and behavioral variables. Only significant correlations between variables were considered part of possible paths (see figure 2). Our results thus allow for five supported paths between morphology through performance to behavior. We performed path analysis on the independent contrasts using the partial least squares method as implemented in the SmartPLS program [56]. Missing data were replaced with variable means (see table 3). All five paths were tested for mediation of performance on the relationship between morphology and behavior using Sobels test of mediation [56], [57]. To this end, path coefficients were calculated with 500 bootstrap replicates with the default settings in SmartPLS. In addition, we tested for mediation of the performance variables on the effect of the morphology variables on the behavioral variables using a model including all variables.

## Results

### Pinch Forces and Morphology

Chela pinch forces were measured on 18 species (table 3). Maximum measured pinch forces, averaged per species, ranged from 0.5N (*Parabuthus transvaalicus*) to 26.4N (*Hetrometrus laoticus*). If these absolute values are corrected for body size by dividing by the square of the prosoma length, we get an index of bite force ranging from 0.0052 (*Parabuthus*) to 0.222 (*Ophistacantus asper*), a range spanning two orders of magnitude.

### Behavior

Each of 231 specimens was given 15 defensive challenges, resulting in a total of 3.465 behavioral responses. Out of these, 528 (15.5%) of the responses did not involve chelae or telson, 212 (6.1%) involved only the chelae, 1.073 (31.0%) involved only the telson, and the remaining 1.642 (47.4%) involved both the chelae and the telson. Table 3 shows the responses per species.

**Figure 3:**
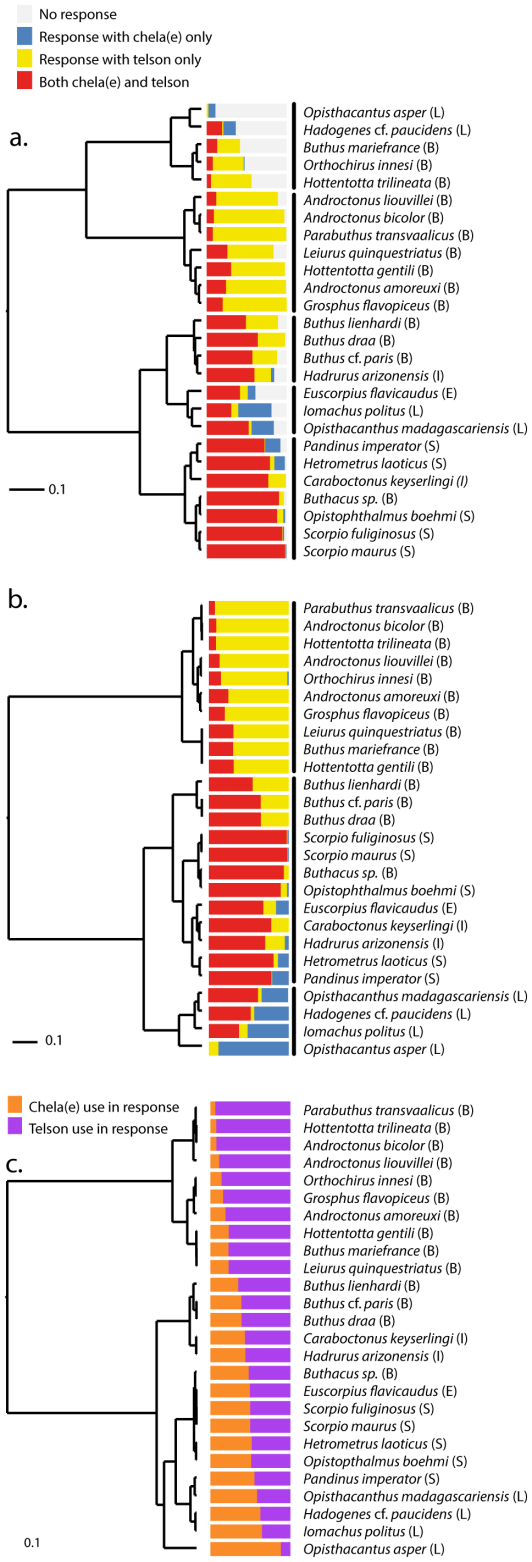
Clustering of species by behavioral response. Clustering based on all response classes (a.), only responses using chelae and/or telson (b.) and proportions of chela and telson use (c.). Colored bars show the proportions of each defensive response class per species.

The proportions of the behavioral response categories per species were clustered for display purposes (figure 3). Very large differences in the proportions of the different response classes can be seen between species. Some species, e.g. *Ophistacantus asper* and *Hadogenes paucidens* also show a high proportion of responses in which neither chelae or telson were used (light grey bars, figure 3a). When we exclude these non-responses, we see that the smaller of the two basal clusters is made up almost entirely of species from the family Buthidae (figure 3b). These species respond mostly by using their telson, which, in less than a third of the responses, is augmented by the use of the chelae. Species comparisons using a Fisher's exact test show no significant differences between these individual Buthidae species (table 2). An exception is the psammophilous buthid *Buthacus,* which clusters with the scorpionids *Scorpio* and *Opistophthalmus*. When the behavioral responses are classified as either using the chelae or the telson (counting the responses using both for both classes; figure 3c) there seems to be a fairly smooth distribution ranging from almost entirely telson-driven responses (*Parabuthus transvaalicus*) to almost entirely chelae-involved (*Ophistacantus asper*).

Since the defensive trials included two different treatments, restricting chelae and prosoma, we tested for differences between these treatments. A Fisher's exact test showed no significant difference between the chelae, but there was a significant difference in the responses to holding down the chelae versus prosoma.

### Phylogenetics

**Figure 4:**
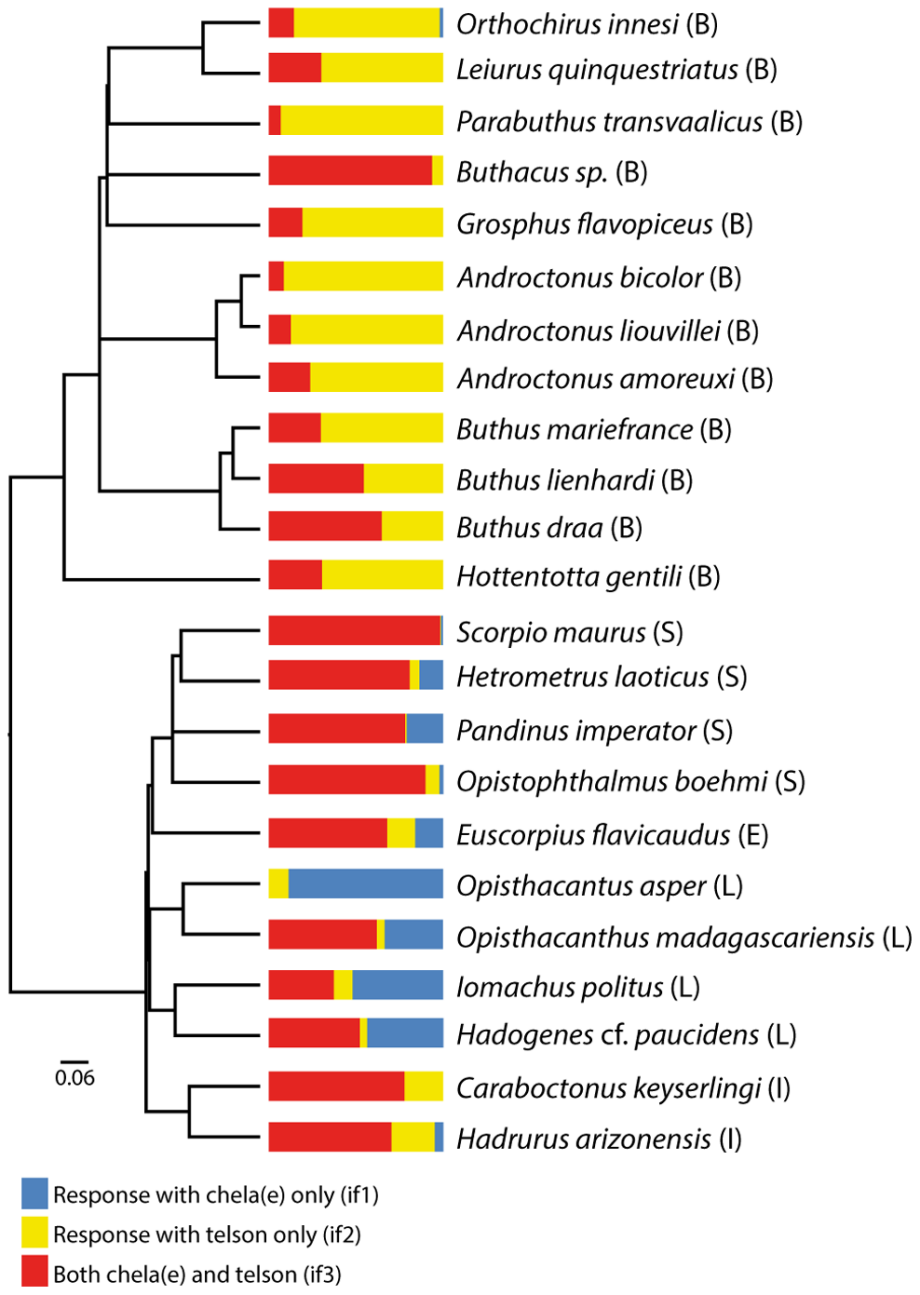
Ultrametric phylogram with branch lengths based on 12S, 16S and CO1 sequences. Colored bars showing proportions of behavioral responses, excluding non-responses, are placed at the tips.

The 12S gene fragment contained 292 parsimony informative sites of a total of 487, the 16S gene fragment contained 39 parsimony informative sites of a total of 181, and the CO1 gene fragment contained 210 parsimony informative sites of a total of 636. Despite their information content, these three genes were not sufficient to resolve all branches among the included taxa with high support. Only the branch uniting all non-buthid taxa received high bootstrap support (99%). The resulting tree (figure 4) was subsequently used in the phylogenetic independent contrasts analysis.

### Non-phylogenetic Correlations

Logistic regressions of behavioral variables on performance and morphological variables showed that relative metasoma length and chela AR were significantly correlated with behavior. Chela AR is correlated with telson use (2) and total chela use (TotC), both when including (2: p = 0.023; TotC: p = 0.044) and excluding non-responses (p = 0.015 for both). When non-responses were excluded, also relative metasoma length showed significant correlation to telson use (p = 0.036) and total chela use (p = 0.036). The LD50 showed significant correlations with several morphological variables: chela AR (slope **−**0.78; p = 0.0082), finger length (0.75; 0.013), mechanical advantage (−0.75; 0.013), relative metasoma length (−0.75; 0.013). The LD50 also showed correlation with chela performance: absolute pinchforce (0.66; 0.038) and relative pinch force (0.69; 0.027). No correlation was found between LD50 and any behavioral variable.

### Phylogenetic Independent Contrasts

The phylogenetic independent contrasts analysis resulted in 22 contrasts, which showed several correlations between morphology, performance and behavior (Table 4). Metasoma AR is positively correlated with chela use (1) and negatively correlated with metasoma use (2). Thus, the evolution of a thicker metasomas is associated with a behavior that favors the use of the stinger in defense rather than the chelae. Interestingly, LD50 co-evolves with relative finger length. Our dataset also shows evolutionary correlation between the two defensive complexes; the relative length of the movable finger correlated significantly with metasoma AR. Sobel’s test of mediation performed on the independent contrast data showed that, of the six paths tested, only the path between chela aspect ratio, maximum force and behavioral category 2 (stinger use) displayed partial mediation of the performance variable (p = 0.026). Two more paths showed indications of mediation, but the effects were found not to be significant (Mech.Adv - Rel. Force - IF1, p = 0.088; Mech. Adv. – Rel. Force – TotT, p = 0.069).

**Table 4:** Regressions of independent contrasts (slope; p-value).

	Rel. force	Metasoma AR	Rel. metasoma L	Mech.Adv.	Chela AR	Rel. finger L	LD50	0	1	2	3	TotC	TotT	if1	if2	if3
Absolute Force	0.67; 0,005	–	–	**−0.15; 0.03**	**−1.22; 0.01**	–	–	0.32; 0.08	–	**−**0.25; 0.08	–	–	–	–	**−**0.24; 0.08	–
Relative Force		–	**−**0.73; 0.08	**−1.55; 0.02**	–	–	–	**3.17; 0.004**	–	–	–	**−**2.22; 0.06	**−2.49; 0.04**	3.18; 0.007	–	–
Metasoma Aspect Ratio			–	–	–	**0.11; 8e-4**	–	–	**0.18; 0.02**	**−0.46; 0.02**	–	–	–	–	–	–
Relative Metasoma Length				0.55; 0.08	–	–	–	–	–	–	–	–	–	**−1.3; 0.03**	–	–
MechanicalAdvantage					**6.98; 1.2e-4**	**−0.27; 0.01**	–	–	–	–	–	–	–	–	–	**−**1.61; 0.09
Chela Aspect Ratio						**−**0.02; 0.06	–	–	–	–	–	–	–	–	–	–
Relative Finger Length							**10.2; 0.015**	–	**1.1; 0.02**	**−**1.99; 0.099	–	–	**−**2.6; 0.09	–	–	–
LD50								–	–	–	–	–	–	–	–	–

Dashes indicate p-values over 0.1. Values with a significance of 0.05 or better are shown in bold face.

## Discussion

We found that in addition to their morphological variation, scorpions are also highly variable in defensive performance and behavior. Our results suggest that evolution in behavior is coupled to both maximum performance and morphology in the case of defensive behavior in scorpions (table 4). This suggests that in situations when survival calls for maximum performance, behavior is correlated with performance. Since the intensity of the simulated attacks in this study was severe, we found a tendency for scorpions to select their most compelling defensive behavior and performance. In species with strong chelae, this corresponds to a relatively higher use of the chelae in defense. However, this does not require that the increased performance of the chelae is due to selection on its defensive use only.

### Behavior

Our data on defensive behavior show that almost half the responses involve both the chelae and telson. This may indicate that the intensity of the perceived attack in the trials was fairly high. Nearly a third of the responses involved use of the metasoma only, and this type of response was much more prevalent in the Buthidae, as was already noted by Warburg [22]. It is noteworthy that the most medically relevant species are in this family (*Androctonus*, *Hottentotta*, *Leiurus*, *Parabuthus*). Chela-only responses are mostly restricted to the Liochelidae and Scorpionidae. Fisher’s tests show that there are clear behavioral differences between many species (Table 2). Our experimental design employed an increasing level of attack intensity, as first the chelae were restrained, followed by restraint of the prosoma. The latter produced a significantly stronger response. However, this difference may also be due to the order in which these treatments were applied as the chela restraint preceded the prosoma restraint in all trials. In addition, restraint of one of the chelae left the scorpion with one less chela to respond with, whereas restraint of the prosoma leaves both chelae and the metasoma free for response. This may have resulted in a lower response rate using the chelae, and therefore a lower overall response intensity. Our results corroborate previous findings [19] that unilateral stimulations elicit a symmetrical defensive response. The behavioral response classification system used here does not encompass all the possible defensive behaviors that a scorpion may use when attacked by a predator. For instance, both violent squirming and escape behavior, undirected chelae and telson movements, as well as total non-responsiveness were all classified as “neither chelae nor telson” (0) in our classification system. Our objective however, was not to obtain a full ethogram of defensive behavior in scorpions, but rather to gain quantitative data on chela and metasoma use in a defensive context. For a more detailed investigation of defensive responses in scorpions, see the works of Palka and Babu, and Warburg [19], [22]. Our choice to make behavioral observations at a constant temperature for all species, rather than at the optimal temperature for each species, may have introduced a bias in the data, as scorpion behavior varies with temperature [58]. However, the alternative, to perform the behavioral observations at the optimum temperature for each species, has practical and systematic limitations. For example, for most species the optimum temperature is simply not known.

### Non-phylogenetic Correlations

Our results show significant correlations between aspects of chela and metasoma morphology and defensive performance. The widely used rule of thumb that species with slender chelae and relatively large metasomas possess more potent venom is corroborated by our data. However, the actual danger that a scorpion poses to a human subject will also highly depend on how much venom a scorpion possesses, and how much gets injected during a defensive sting. Scorpions are known to meter their venom, as venom production places a high demand on the energy budget [59], [60].

### Phylogenetics

The three mitochondrial genes used for phylogenetic reconstruction proved insufficient to resolve the phylogeny with good support. Although well-supported branches did not contradict current understanding of scorpion systematics, most internal branches lacked support. In this study, lack of resolution forced us to introduce several polytomies. However, despite the decrease this caused in the number of degrees of freedom in the phylogenetic independent contrasts analysis, we detected significant correlations between morphology, performance and behavioral parameters.

### Phylogenetic Independent Contrasts

The phylogenetic independent contrasts analysis showed that there exist several significant correlations between morphology, performance and defensive behavior (table 4). The correlation between pinch force versus mechanical advantage can be understood as the direct mechanical consequence of chela design on performance. A negative correlation between mechanical (displacement) advantage and force is expected if the chela mechanical design is optimized for force production. A similar rationale can be made for the negative correlation with chela AR and force; a lower AR chela allows for more muscle to be packed into the manus. In addition, these low AR chela designs seem to also lower the stress in the cuticula under maximum pinch performance [18]. Also, we expect that morphological aspects of the same structure can evolve in concert, either due to physical dependence, developmental constraints or a common selection pressure. We found e.g. that the chela AR significantly correlated with mechanical advantage. The correlations between morphological aspects of the two defensive systems however, cannot be explained by direct mechanical, spatial or developmental consequences. The chelae and metasoma are at opposite sides of the animal, and therefore will not correlate due to direct spatial interaction. Moreover, since the chelae are parts of the appendages, and the metasoma consists of the last five segments of the body, they are unlikely to correlate due to common developmental pathways. However, genetic correlations (i.e. pleiotropy) other than common developmental pathways could still account for correlation in these traits. We found that possessing relatively longer fingers on the chela is associated with thicker metasomas. This relationship suggests a trade-off between the metasoma and the chelae. Trade-offs between different organ systems have been described in other organisms, for instance between systems for locomotion and reproduction [61], [62]. Whether this trade-off is due to different selective optima in a defensive context, or due to other evolutionary constraints, remains unresolved. The chelae and metasoma are both multifunctional, and are used in other behaviors, such as sensing the environment [63], and mating [64]. The apparent trade-off between chelae and metasoma may be driven by optimization for another function, and differences in defensive response may only reflect the selection of the most compelling dissuasion stimulus to a predator.

Although the morphological aspects of both defensive systems could be related to behavior, only the performance of the chelae could be linked to behavior. In contrast, LD50 was not correlated with behavior. Yet, this may possibly be due to the limited number of species for which these values could be found in the literature. Studies on *in-vivo* venom potency are limited as current regulations on ethics in animal testing often do not allow LD50 tests to be performed on live mice, and alternative methods (e.g. [65]) have not found broad acceptance yet. We therefore were forced to select closely related species when these were available. Although not formally tested, closely related species often share a similar venom potency. In addition, different methods of extraction, purification and injection employed in the different studies may have increased the noise to data ratio in the LD50 dataset. An investigation in the defensive performance using venom should therefore not only include venom metering, but also standardized methods of obtaining in-vivo efficacy of venoms in non-animal or non-sentient systems.

In one case, path analysis recovered partial mediation of performance on the relationship between morphology and behavior. Interestingly, performance of the pincers (maximum pinch force) mediated the relationship of chela morphology (AR) on defensive stinger usage (2). Since the relationship between pinch force and stinger usage is negative, this indicates that chela design allows for a larger pinch force, which negates the necessity to use the stinger in defense. Or, conversely, the development of a deterrent venom reduced the necessity for strong chelae. A more well-resolved and complete phylogeny of scorpions will be necessary to address the causal direction of this relationship.
